# Ultra-Processed Food Availability and Noncommunicable Diseases: A Systematic Review

**DOI:** 10.3390/ijerph18147382

**Published:** 2021-07-10

**Authors:** Taissa Pereira de Araújo, Milena M. de Moraes, Vânia Magalhães, Cláudia Afonso, Cristina Santos, Sara S. P. Rodrigues

**Affiliations:** 1 Faculty of Nutrition and Food Sciences, University of Porto, 4150-180 Porto, Portugal; milenaferreira@fcna.up.pt (M.M.d.M.); claudiaafonso@fcna.up.pt (C.A.); cristinasantos@fcna.up.pt (C.S.); saraspr@fcna.up.pt (S.S.P.R.); 2 Public Health Institute, University of Porto, 4050-091 Porto, Portugal; vaniaplmagalhaes.st@gmail.com; 3 Faculty of Medicine, University of Porto, 4200-319 Porto, Portugal; 4 Center for Health Technology and Services Research, University of Porto, 4200-450 Porto, Portugal

**Keywords:** food processing, households, noncommunicable diseases, systematic review, ultra-processed food

## Abstract

Ultra-processed food (UPF) can be harmful to the population’s health. To establish associations between UPF and health outcomes, food consumption can be assessed using availability data, such as purchase lists or household budget surveys. The aim of this systematic review was to search studies that related UPF availability with noncommunicable diseases or their risk factors. PRISMA guidelines were used. Searches were performed in PubMed, EBSCO, Scopus and Web of Science in February 2021. The search strategy included terms related to exposure (UPF) and outcomes (noncommunicable diseases and their risk factors). Studies that assessed only food consumption at an individual level and did not present health outcomes were excluded. Two reviewers conducted the selection process, and a third helped when disagreement occurred. The Newcastle–Ottawa Scale was used to assess the studies’ quality; 998 records were analyzed. All 11 eligible studies were ecological and assessed overweight and obesity as a health outcome, only one showed no positive association with UPF availability. Two studies included the prevalence of diabetes as an outcome, however no significant association was found with UPF availability. Studies relating UPF availability and health outcomes are focused on overweight and obesity. It is necessary to further explore the relationship between other health outcomes and UPF availability using purchase or sales data.

## 1. Introduction

Over time, changes in the food environment lead to modifications in the population’s health [1]. Broad changes in dietary and physical activity patterns, obesity trends and diet-related non-communicable diseases (NCDs) are part of the concept of nutritional transition, of which the fourth stage emphasizes the change from the consumption of processed to ultra-processed food (UPF) [2,3,4].

UPF are described as “processed ingredients typically combined with the sophisticated use of additives to make them edible, palatable and habit forming” [5,6,7]. Although this definition dates from 2010, before this, these foods were already referred to in the literature, either as an independent food group or as particular foods. Food processing often leads to increased nutrient bioavailability, either for beneficial (lycopene from tomato) or deleterious effect, such as the increase in the proportion of sugars [8,9]. Processed and UPF are generally less satiating than fresh foods [10,11].

Reference reports have stated that consumption of processed foods and drinks such as “fast food”, “convenience foods”, soft drinks, sugary drinks, processed meat and others is associated with obesity and several chronic non-communicable diseases [1,12]. The latest updates to dietary guidelines recommend a decrease in the frequency of UPF consumption. The updated Mediterranean Dietary Pyramid (MDP), which represents environmental sustainability, has processed meat and sweets on the top, suggesting that they should only be consumed occasionally [13,14,15].

The guidelines for the collection of information on food processing through food consumption surveys indicate that such information can be used for different purposes, including assessment of the relationship between food processing and obesity and NCDs and monitoring time trends in the consumption of processed foods [16]. The information sources for studying such relationships need to be based on dietary data, which may be evaluated either by using estimates based on direct or indirect assessment methods. Direct methods include 24-h recall, food frequency questionnaires and other individual-based dietary assessment. Individual-level assessments measure food intake, while indirect methods refer to food supply or availability, usually estimated at national or household level [17]. For example, expenditures or acquisition lists, such as data derived from household budget surveys, can be an extremely important instruments to assist in long-time assessment of UPF availability [18,19]. The lack or scarcity of individual dietary surveys in many countries makes these indirect data the only tool available to assess food consumption and study time trends. Such a fact exposes the relevance of identifying whether the associations observed when using individual data are maintained when an ecological approach is used. Although there are several recent systematic reviews on UPF consumption and health outcomes [20,21,22,23,24,25,26], there are none that focus on the availability of UPF with health outcomes.

Thus, the aim of this review was to compile and analyze studies that related UPF availability with mortality and morbidity from NCD diseases or their risk factors.

## 2. Materials and Methods

This study was submitted to the International Prospective Register of Systematic Reviews—PROSPERO (CRD42020162978).

### 2.1. Data Sources and Search Strategy

The PRISMA (Preferred Reporting Items for Systematic Reviews and Meta-Analyses) [27] guidelines were followed. To identify primary studies, searches in four electronic bibliographic databases were performed: PubMED, EBSCO Academic Search Ultimate, Scopus and Web of Science. The search strategy was related to exposure (foods according to food classification systems based on processing) and outcomes (mortality, incidence, and prevalence of NCDs and their risk factors) of interest. The search terms were defined through an exploratory investigation to identify keywords, in addition to the Medical Subject Headings (MeSH). Search terms were: [((ultraprocessed OR ultra-processed OR “highly processed”) AND food*) AND (risk OR factors OR predictors OR determinants OR prevalence OR incidence OR change OR association) AND (“noncommunicable diseases” OR “chronic diseases” OR metabolic syndrome OR obesity OR overweight OR diabetes OR cardiovascular OR cancer OR dyslipidemia OR health OR morbidity OR mortality)]. Only “noncommunicable diseases” was used because no differences were found while also using “non-communicable diseases”. The search was last updated on February 2021, with no limits for time or language. Two independent reviewers assessed the eligibility of the selected papers. In the first step, the studies were selected based on the title and abstract. In the second stage, the full text of the articles was read. Backward citation tracking was used to find other relevant studies. A grey literature search was performed in the OpenGrey database.

### 2.2. Eligibility Criteria

The study types to be included were intervention and observational studies. As an inclusion criterion, it was necessary that the study was based on UPF availability data and its relationship with a health outcome. The exposure variable was the availability of UPF. The outcome variable was any observed measure related to health. Studies that assessed only food consumption at the individual level and that did not present a linkage with health outcomes were excluded.

### 2.3. Data Extraction

Two reviewers selected the studies. A third reviewer participated when disagreement on the studies’ selection occurred. The following information was extracted from each study: author (publication year); country; study design; data source (research year); exposure variables; outcome variables; statistical analysis method; main results.

### 2.4. Quality Assessment

To evaluate the quality of the selected studies an adapted version of the Newcastle–Ottawa assessment scale for non-randomized study designs was used [28,29]. A seven-question checklist was filled in for eligible studies by two independent reviewers. The maximum score that could be attained was 10 points.

## 3. Results

**Figure 1:**
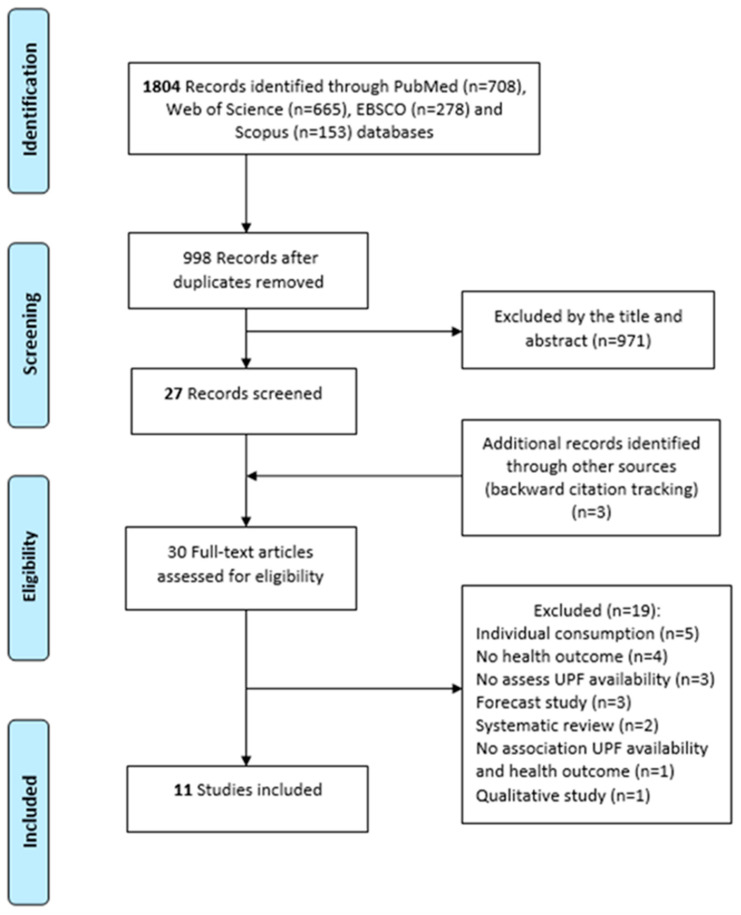
Flowchart of the study selection for the present systematic review.

The search found 1804 studies (708 from PubMed, 665 from Web of Science, 278 from EBSCO and 153 from Scopus). Duplicates were removed and 998 titles and abstracts were analyzed. After the screening, the three reviewers agreed to read 27 studies in full, all of them in the English language. Two reviewers agreed regarding the selection of six and disagreed on four studies. Again, the third reviewer participated and agreed that two of these four articles were relevant. Eight articles were eligible to be included. Three eligible studies were found with backward citation tracking and no other articles were located through the grey literature search (Figure 1).

From the final 11 eligible studies, six were multi-country approaches and the others were performed in Brazil (n = 3), Guatemala (n = 1) and Sweden (n = 1). All studies used an ecological design (n = 11). Five of them were cross-national times series. All had representative samples, five were national samples, and the others were based on particular population subgroups (Table 1).

According to the exposure variable, the studies assessed the availability of UPF in general (n = 7) or of isolated UPF in particular (n = 4). Of the studies that evaluated isolated UPF, three evaluated the availability of only sugar sweetened beverages (SSB) and one evaluated the availability of fast food (Table 1).

The outcomes found in the selected studies included overweight (n = 8), obesity (n = 7), BMI (n = 6) and prevalence of diabetes (n = 2) (Table 1). The two studies that assessed the prevalence of diabetes as a health outcome evaluated only a particular type of UPF (SSB) (Table 1).

Most of the studies observed a positive association with BMI, overweight and obesity. Only one showed no positive association between UPF availability and overweight but a smaller variety of available vegetables was associated with overweight. However, no significant association was found with diabetes (Table 1).

Quality evaluation scores ranged between four and nine, out of ten possible points. Seven articles obtained a score of six (Table 2).

## 4. Discussion

Of the 11 articles, six evaluated availability through purchase and five through sales of UPF. These five studies used the Euromonitor Passport Global Market Information Database, which collects sales volume data, from many countries worldwide, from various sources including trade associations, industry bodies, company financial reports, and official government statistics [31,33,35,38,39]. The others six studies in this systematic review used purchase data from HBS [30,32,34,36,37,40]. To classify food according to the purpose and extent of processing, six studies [30,32,33,34,36,37] used NOVA [7], a system that was launched in 2010, and one used a classification developed at the International Food Policy Research Institute [40]. The other four specified the type of UPF studied, namely, soft drinks [31,35,39] and fast food [38].

Almost all studies in this systematic review showed a positive association or correlation between UPF availability and increase of BMI, overweight or obesity, only one showed no significant association between UPF availability and overweight. However, the only study that did not find a positive association used a biased sample [30]. Although the study defined the population as representative of a municipality, in fact, the population was from primary health care services that had access to health intervention programs [30].

Of the 11 selected studies, five studies explored the association of UPF availability with only BMI increase [33,38], overweight [30,32] or obesity [34]. The other six studies evaluated the association of UFP availability using both prevalence of overweight or obesity as separate outcomes in the same sample [31,35,36,37,39,40]. Three of them studied soft drink sales [31,35,39]. Basu et al. [39] showed that the soft drink consumption was strongly associated with overweight and obesity. Both Ferretti and Mariani [31] and Goryakin et al. [35] showed that the increased availability of UPF was similarly associated with the increased prevalence of overweight and obesity. Juul and Hemmingsson [36] evaluated overweight and obesity time trends separately, and concluded that the increasing trends followed the increase in UPF availability. However, due to the study methods used, the association between the outcome and exposure was not calculated. Canella et al. [37] and Asfaw [40] followed the findings of the other studies. Canella et al. [37] evaluated mean BMI, overweight and obesity, and found a positive association with increase in UPF availability. Asfaw [40] found that an increase in the share of highly processed foods (in the total food expenditure) significantly increases the likelihood of overweight and obesity.

Only two articles related the availability of UPF with other health outcomes, in this case diabetes, and did not find a significant association [35,39]. These two articles have only SSB as an exposure variable. These findings may reflect residual confounding. Basu et al. [39] described a limitation of their study as the fact that the soft drink consumption data did not include fruit drinks (fruits and vegetable juices), which have been independently related to the risk of diabetes, likely because of their high sugar content. Goryakin et al. [35] reported that their soft drink price models might suffer from potentially important unobserved confounding, because they assumed that soft drink sales/prices affect BMI and diabetes with only a one-year lag.

Four studies that used UPF availability data were excluded, as they did not evaluate health outcomes. Three studies used UPF availability data for forecast estimation [41,42,43], two studies analyzed dietary patterns [44,45] and one was a qualitative study [46]. Qualitative studies do not measure the relationship between UPF and health outcomes, but are useful to explore topics and suggest interventions [46].

All eligible studies were ecological. No specific quality assessment was found in the literature for this type of study. However, the Newcastle–Ottawa quality assessment scale adapted for cross-sectional studies was used. However, the lack a specific scale may lead to under or overestimation of final scores. Based on the Newcastle–Ottawa adapted scale, most studies showed positive results: all of them presented a representative sample, with a justified and satisfactory size. Nine studies reached the maximum score in regard to comparability, such as the control of confounding factors [30,31,33,34,35,37,38,39,40]. Only one study did not score on the statistical test [36]. Only two studies obtained a score in the description assessment of outcome [30,37]. Only one study obtained a maximum score for the ascertainment of exposure [30]. None of the studies presented a description of the non-respondents’ characteristics.

### Limitations and Strengths

The first limitation of this review was the variability of methods used to investigate food availability, either acquisitions/purchases or sales. This factor hindered evaluation of the data by means of meta-analysis. A second limitation was related to the fact that the selected articles were not uniform with each other regarding the exposure measure, for example, using different UPF classifications or only a specific UPF. Another limitation was that most of the studies analyzed only BMI and the prevalence of overweight and obesity. In addition, a possible publication bias might be expected, since only one of the studies showed no significant association with health outcomes.

The main strength of this review is its broad approach, including reference lists as well as grey literature, and not restricting language or publication date. In addition, the clear identification of inclusion and exclusion criteria and the presence of three reviewers reduced possible bias. Despite the fact that all the eligible studies were ecological, many of them used HBS food availability as the base of the exposure variable. Although national studies based on individual consumption show results with greater consistency, they are scarce and not comparable in many countries. On the other hand, HBS have a periodicity of data collection with similar methodology among countries and have been recognized as a highly cost-effective tool for monitoring food patterns [47,48]. Therefore, these data are an important source for studying associations between food consumption and health outcomes [20,41]. Results from longitudinal studies produce more robust evidence [49,50], however, such studies are more long-lasting and expensive than ecological studies. In this systematic review, six authors performed temporal series studies either with HBS or sales data [33,34,35,36,38,39]. This study design allows assessment of the changes that occur over several periods unlike a single cross-sectional study. This research shows that the results for the availability of UPF associated with overweight and obesity follow the same trend as cross-sectional and longitudinal studies that use individual dietary data [51,52].

## 5. Conclusions

Studies with UPF are recent but evidence of the association of UPF with NCDs has been observed in both the studies shown in this systematic review and other systematic reviews that assess consumption through individual data. Scientific papers that assess the availability of UPF and health outcomes have prioritized the relationship with BMI or overweight and obesity, showing evidence of their positive association. Availability data are effective for trend studies, and are often the only data available to assess consumption. This suggest the relevance of further exploring the availability data to assess the relationship between UPF and other health outcomes, such as incidence, prevalence and mortality from cardiovascular diseases, diabetes or cancer.

## Figures and Tables

**Table 1:** Summary of the eleven studies included in the systematic review of UPF availability associated with health outcomes (February 2021).

Author (Publ. Year)	Country	Study Design	Data Source (Study Year)	Exposure Variables	Outcome Variables	Statistical Analysis Method	Main Results
de Freitas et al. (2019) [30]	Brazil (Belo Horizonte)	Ecological Cross-sectional	Sample representative of the municipality: 2810 participants in 18 Health Academy Program (HAP) units selected by stratified cluster sampling according to 9 administrative regions of the city (2013)	Availability of fruits and vegetables and UPF on consumer food environment—all retail stores and open-air food markets located within 1600 m of each HAP unit	Prevalence of overweight	Multivariable multilevel logistic regression	No significant association between UPF availability and overweight. A smaller variety of vegetables was associated with overweight
Ferreti and Mariani (2019) [31]	150 countries worldwide	Ecological	Data on per capita sugar-sweetened beverage (SSB): Euromonitor ** 2012 edition. Age-standardized mean BMI: Global Burden of Metabolic Risk Factors of Chronic Diseases Collaborating Group (2014–2015)	Percentage of the consumer’s income (measured by the gross national income per capita) required to buy 100 L of SSB	Prevalence of overweight and obesity	Multivariate regression	Positive association between increase in SSB affordability (10%) and overweight (0.4 more adults per 100) and obesity (0.38 more cases per 100 adults).
Vale et al. (2019) [32]	Brazil	Ecological	Household Budget Survey (POF) and National Household Sample Survey (PNAD): Brazil’s 27 states (national representative sample). POF: each household is assigned a sampling weight from which the estimates of prevalence of excess weight are obtained. (2008–2009)	Proportion of unprocessed or minimally and ultra-processed foods (according to the NOVA classification.) on annual per capita household food purchases in kg	Prevalence of the excess weight	Spatial analysis: correlation	A moderate correlation between purchase of ultra-processed foods and prevalence of excess weight (*p* = 0.01).
Vandevijvere et al. (2019) [33]	80 countries	Ecological	Data on total volume sales of foods and drinks per capita: Euromonitor ** (80 countries) Food subgroups: NOVA classification. BMI data: NCD-RisC group (all countries). (2002–2016)	Total volume sales of ultra-processed drinks (UPD) and ultra-processed foods (UPF) *per capita* summed by country and year.	Mean BMI *	Mixed models for repeated measures: spatial power covariance structure	Positive association between total increase of UPF and UPD sales and increase of mean BMI for both men and women.
Monteiro et al. (2018) [34]	19 European countries	Ecological	UPF household availability data from Data Food Networking (DAFNE): European data depository of national household budget surveys; and the Living Costs and Food Survey (for UK data) National representative samples. (1991–2008)	National average daily per capita availability of NOVA food groups expressed as percentage of total purchased dietary energy.	Prevalence of obesity	Linear regression	Significant positive association between national household availability of UPF and prevalence of obesity in adults. Each 1% increase of UPF household availability = 0.25% increase in obesity prevalence (r = 0.63; 95% CI = 0.05, 0.45; *p* < 0.02)
Goryakin et al. (2017) [35]	78 countries	Ecological (cross-national time series)	Data on soft drink sales: Euromonitor ** 2014 edition. Data on age-standardized country-level BMI levels, overweight, obesity and diabetes prevalence: NCD-RisC group. (1999–2014)	Per capita sales of carbonated soft drinks derived by dividing the off trade volume of these drinks by the population of each country.	Mean BMI *, overweight, obesity and diabetes prevalence.	Longitudinal panel analyses; Multivariate regression models of fixed effects	Trends of increase soft drink sales per capita have been accompanied by an increase in mean BMI and in average overweight and obesity prevalence. Soft drink sales were unrelated to diabetes prevalence.
Juul and Hemminssong (2015) [36]	Sweden	Ecological	Food availability data: Swedish Board of Agriculture (4000 randomly selected households—national representative sample). Overweight and obesity data: nationwide database of Statistics Sweden and the WHO Global Health Observatory Data Repository (+18 years). (1960–2010)	Per capita availability of unprocessed or minimally processed foods, processed culinary ingredients, processed and ultra-processed foods (NOVA system)	Mean BMI *, prevalence of overweight and obesity	Time-trend descriptive analysis	Trends of increase UPF availability have been accompanied by an increase in overweight and obesity prevalence.
Canella et al. (2014) [37]	Brazil	Ecological	Complex clustered sampling procedure, first selecting census tracts and then selecting households within those tracts.National representative sample: 55,970 households. (2008–2009)	Purchase data of all foods and drinks for home consumption, expressed in daily kilocalories (kcal) per capita, classified into 3 groups: fresh or minimally processed foods, processed culinary ingredients, processed or ultra-processed food (UPF).	BMI * and Z-scores of BMI-for-age (≤19 y). Prevalence of excess weight and obesity	Linear regression	Positive and independent association between household availability of UPF (1st to 4th quartile) and BMI z-score (0.53–0.81), excess weight (35.6–41.7%) and obesity (9.9–13.6%).
De Vogli et al. (2014) [38]	25 countries ***	Ecological (cross-national time series)	Data on per capita fast food transactions: Euromonitor ** 2012 edition. Age-standardized mean BMI: Global Burden of Metabolic Risk Factors of Chronic Diseases Collaborating Group (1999–2008)	Industry records of annual sales of meals and refreshments delivered in local and transnational fast food outlets, including chain restaurants, independent eateries and convenience stores.	Mean BMI *	Longitudinal panel analyses; Multivariate regression models of fixed effects	Positive association between increase in annual fast food transactions (1-unit per capita) and increase in age-standardized mean BMI (0.033 kg/m^2^ 95% CI: 0.013–0.052). Only the intake of soft drinks mediated the observed association (β: 0.030; 95% CI = 0.010–0.050).
Basu et al. (2013) [39]	75 countries	Ecological (cross-national time series)	Data on soft drink sales: Euromonitor ** 2011 edition. Age-standardized overweight prevalence data: World Health Organization’s Global Database on BMI (2011 edition). Diabetes data: International Diabetes Federation. (1997 to 2010)	Per capita annual sales of carbonated soft drinks in gallons, including both imported drinks and those manufactured domestically	Prevalence of overweight, obesity and diabetes	Multivariate linear regression	Strong and positive correlate with the prevalence of overweight (r = 0.62; *p* < 0.001) and obese adults (r = 0.55; *p* < 0.001). Increase in soft drink consumption (1%) was associated with an additional 4.8 overweight and 2.3 obese adults per 100 (95% CI = 3.1, 6.5; 1.1, 3.5), and 0.3 adults with diabetes/100 (95% CI = 0.1, 0.8)
Asfaw (2011) [40]	Guatemala	Ecological	National representative sample: 7276 households (38 municipalities in 22 departments and eight regions) (2000)	Per capita value of meals consumed outside home and per capita total food expenditure with unprocessed, primary processed and highly processed food	BMI *, prevalence of overweight/obesity	Generalized method of moments regression	Positive association between increase of highly processed food expenditure (10%) and increase of body mass index—BMI (4.25%).

* BMI = Body index mass (in kg/m^2^); Excess weight or Overweight = BMI ≥ 25 kg/m^2^ (or BMI ≥ 27 kg/m^2^ if >60 years); Obesity = BMI ≥ 30 kg/m^2^. ** Euromonitor’s Passport Global Market Information Database: Representative sample of online consumers in each country. *** High-income countries from Organisation for Economic Co-operation and Development (OECD).

**Table 2:** Quality assessment.

	Selection (Max. 5)				Comparability (Max. 2)	Outcome (Max. 3)			
	Representativeness of the Sample (Max. 1)	Sample Size (Max. 1)	Non-Respondents (Max. 1)	Ascertainment of Exposure (Max. 2)	Comparable Subjects in Different Outcome Groups. Confounding Factors Controlled.	Assessment of Outcome (Max. 2)	Statistical Test (Max. 1)	Score	Maximum Score
de Freitas P.P. et al. (2019) [30]	1	1	0	2	2	2	1	9	10
Ferreti F. and Mariani M. (2019) [31]	1	1	NA	1	2	0	1	6	9
Vale D. et al. (2019) [32]	1	1	0	1	0	0	1	4	10
Vandevijvere S. et al. (2019) [33]	1	1	NA	1	2	0	1	6	9
Monteiro C.A. et al. (2018) [34]	1	1	0	1	2	0	1	6	10
Goryakin Y. et al. (2017) [35]	1	1	NA	1	2	0	1	6	9
Juul F. and Hemminssong E. (2015) [36]	1	1	0	1	1	0	0	4	10
Canella D.S. et al. (2014) [37]	1	1	0	1	2	2	1	8	10
De Vogli R. et al. (2014) [38]	1	1	NA	1	2	0	1	6	9
Basu S. et al. (2013) [39]	1	1	NA	1	2	0	1	6	9
Asfaw A. (2011) [40]	1	1	0	1	2	0	1	6	10

## Data Availability

All data are presented in the current manuscript (text, tables).
